# Sexual life satisfaction and its associated socio-demographic and workplace factors among Chinese female nurses of tertiary general hospitals

**DOI:** 10.18632/oncotarget.17664

**Published:** 2017-05-07

**Authors:** Feng Ji, Deguo Jiang, Xiaodong Lin, Wei Zhang, Weifang Zheng, Ce Cheng, Chongguang Lin, Lirong Hu, Chuanjun Zhuo

**Affiliations:** ^1^ School of Mental Health, Jining Medical University, Jining, China; ^2^ Department of Psychological Medicine, Wenzhou Seventh People's Hospital, Wenzhou, China; ^3^ Department of Psychological Medicine, Tianjin Anding Hospital, Tianjin, China

**Keywords:** nurse, sexual life satisfaction, occupational factor

## Abstract

Adverse workplace factors such as job stress are reported to be associated with poor physical and mental health of nurses. However, associations between occupational factors and sexual life satisfaction (SLS) of nurses remain understudied. This study investigated SLS of Chinese female nurses of tertiary general hospitals and socio-demographic and occupational factors associated with reduced SLS of nurses. In this cross-sectional survey, 393 Chinese female nurses of four tertiary general hospitals completed a standardized socio-demographic and occupational characteristics questionnaire, Zung's Self-rating Scale for Depression, Job Content Questionnaire, and a self-report SLS question. Multiple ordinal logistic regression was used to identify factors related to reduced SLS. Fourteen point five percent female nurses were dissatisfied with their current sex lives. In multiple regression, related factors for decreased SLS included being unmarried (OR = 1.49), shift work (OR = 1.92), contract employment (OR = 1.63), high job demands (OR = 2.21), low job control (OR = 1.88), inadequate social support (OR = 2.32), and depression (OR = 3.14). Chinese female nurses of tertiary general hospitals have poor SLS. Reducing job stress and providing psycho-social support may help improve SLS of nurses.

## INTRODUCTION

In recent years, there has been an increasing focus on the etiological role of workplace psychosocial factors in the occurrence and development of physical and mental health problems in various occupational populations [1]. Nurse is a typical occupational population that provides nursing services to patients in medical institutions, whose work is characterized by high demands for nursing skills, heavy laboring intensity, and high risk of occupational exposure, making nurse at high risk for job stress [2]. Until now, there have been many studies reporting the significant relationships of adverse work-related factors (i.e., extended hours of working and job stress) with poor physical and mental health of nurses [3–7]. Negative work-related factors also may adversely affects sex function. A few studies has reported that night-shift work, longer working hours, and “the pressure to do more in less time” had significant deleterious effects on sexual health of both males and females [8–12]. Nevertheless, very few studies have focused on workplace factors influencing sexual health of nurses.

Sexual life satisfaction (SLS) is a comprehensive measure of sexual function, which is defined as the degree to which an individual is satisfied or happy with the sexual aspect of his or her relationship [9, 13]. Having a satisfactory sexual life is crucial for maintaining a good quality of life because it is the basis of a happy marriage and family and an essential component of health-related quality of life [14–18]. One study has reported the poor marital quality of Chinese nurses and worsened sexual life as one of its most important causes [16].

In contemporary China, nurses of large general hospitals often work under very high pressure due to various types of work-related stressors and the growing risk of hospital violence against medical personnel [19, 20]. Given the association between sexual dysfunction and workplace factors, Chinese nurses may have poor SLS. However, the epidemiology of SLS in Chinese nurses remains largely unknown. Understanding the SLS and its associated workplace factors, would facilitate efforts to promote the quality of life in Chinese nurses. The present study examined the prevalence and correlates of SLS among Chinese nurses working in large general hospitals.

## RESULTS

The average age of the 393 female nurses was 32.5 years (standard deviation [SD] = 7.9, range = 18–56), 53.4% had an educational attainment of 4-year college and above, 87.3% were married, 37.4% were engaged in shift work, and 73.3% had worked for at least 8 years. The mean job demands, job control, and social support subscale scores of Job Content Questionnaire (JCQ) were 13.8 (SD = 2.1), 25.4 (SD = 2.1), and 16.6 (SD = 3.0), respectively. The average SDS score was 42.7 (SD = 9.5). Other socio-demographic and occupational characteristics of nurse are shown in Table 1.

**Table 1:** Characteristics of nurses and SLS scores by grouping variables

Variables		No. of nurses	SLS score	t	*P*
Age*	< 30 years	227	3.49 ± 0.85		
	≥ 30years	166	3.33 ± 0.96	1.702	0.090
Education	3-year college and below	183	3.22 ± 0.99		
	4-year college and above	210	3.56 ± 0.84	3.642	< 0.001
Marital status#	Unmarried	50	3.20 ± 0.96		
	Married	343	3.49 ± 0.87	2.173	0.030
Self-rated family income	Moderate and good	282	3.50 ± 0.88		
	Poor	111	3.22 ± 0.98	2.748	0.006
Religious belief	No	369	3.39 ± 0.93		
	Yes	24	3.54 ± 0.72	0.768	0.443
Working years*	< 8	105	3.48 ± 0.95		
	≥ 8	288	3.37 ± 0.91	1.091	0.276
Type of work	Shift	147	3.25 ± 0.97		
	Regular	246	3.53 ± 0.88	0.937	0.004
Technical title	Nurse and Nurse Practitioner	285	3.25 ± 0.95		
	Nurse-in-charge and above	108	3.49 ± 0.89	2.274	0.023
Type of employment	Permanent	188	3.51 ± 0.92		
	Contract	205	3.30 ± 0.90	2.257	0.025
JCQ scores					
Job demands*	≥ 15	198	3.23 ± 0.96		
	< 15	186	3.62 ± 0.82	4.281	< 0.001
Job control*	< 26	149	3.24 ± 0.96		
	≥ 26	244	3.66 ± 0.79	4.638	< 0.001
Social support*	< 16	229	3.13 ± 1.01		
	≥ 16	164	3.59 ± 0.79	4.838	< 0.001
Depression	Yes	200	3.11 ± 0.92		
	No	193	3.70 ± 0.80	6.701	< 0.001

Note: *These continuous variables were dichotomized at the median value; # “Unmarried” included never married, cohabitating, and divorced, and ”Married” included married and remarried.

The mean SLS score was 3.4 (SD = 0.9). In total, 14.5% nurses were dissatisfied with their sex lives (3.3% “very dissatisfied” and 11.2% “dissatisfied”), 36.4% rated their sex lives as “fair”, and 49.1% were satisfied (40.2% “satisfied” and 8.9% “very satisfied”).

Results of comparisons of SLS scores between subgroups according respondent characteristics (Table 1) showed that, nurses who had an educational attainment of 3-year college and below, were unmarried, rated their family income level as “poor”, were shift workers, held a technical title of Nurse or Nurse Practitioner, were contract employee, scored high on JCQ-demands subscale, scored low on JCQ-control subscale, scored low on JCQ-social support subscale, and had clinically significant depressive symptoms, were significantly more likely to have low SLS scores.

Multivariable ordinary logistic regression analysis (Table 2) demonstrated that being unmarried (odds ratio [OR] = 1.49), shift work (OR = 1.92), contract employment (OR = 1.63), high job demand (OR = 2.21), low job control (OR = 1.88), inadequate social support (OR = 2.32), and depression (OR = 3.14) were significantly and independently associated with reduced SLS.

**Table 2:** Multivariable ordinary logistic regression on factors associated with reduced SLS of nurses

Factor	Risk group	Reference group	Coefficient	Standard error	Wald χ^2^	*P*	OR (95% CI)
Marital status	Unmarried	Married	0.402	0.204	3.882	0.021	1.49 (1.01,2.23)
Type of work	Shift	Regular	0.654	0.237	7.597	0.006	1.92 (1.21,3.06)
Type of employment	Contract	Permanent	0.486	0.231	4.426	0.018	1.63 (1.18,5.70)
JCQ							
Job demands	≥ 15	< 15	0.794	0.199	15.892	< 0.001	2.21 (1.50,3.27)
Job control	< 26	≥ 26	0.631	0.209	9.067	0.003	1.88 (1.25,2.83)
Social support	< 16	≥ 16	0.843	0.226	13.965	< 0.001	2.32 (1.49,3.62)
Depression	Yes	No	1.143	0.207	30.508	< 0.001	3.14 (2.09,4.70)

## DISCUSSION

It is generally recognized that SLS is the result of multiple physical, psychological, and social factors, but SLS of a special population is additionally influenced by the population‘s specific characteristics [14, 15, 21–23]. Therefore, in addition to showing SLS of Chinese nurses, our study paid special attention to workplace factors of SLS of the nurse population when considering its associated factors. To the best of our knowledge, this is the first study in China that determined the epidemiology of SLS in Chinese nurses working in large general hospitals. The main finding of this study is that 14.5% and 49.1% nurses reported dissatisfied and satisfied with their current sex lives, respectively. Compared to studies using the same item about SLS, this level of dissatisfaction is higher than those of married Chinese women of childbearing age (10%) and Chinese civil servants (5.8%) [24, 25], while this level of satisfaction is lower than that of Chinese urban residents (62.6%) [26]. These comparisons indicate that the level of SLS of Chinese nurses is lower than the Chinese general population.

Multiple regression analysis showed that being unmarried and depression were significant risk factors for decreased SLS of nurses; both findings are consistent with those reported in female heroin-dependent patients, hearing disabled people, older adults, and urban residents [14, 22, 26, 27]. The low SLS in unmarried nurses is expected, because unmarried individuals generally have difficulties in satisfying their sexual desires due to the lack of stable sex partnerships [21]. There is evidence that depressive emotion plays an important role in the etiology of sexual dysfunction and sexual dysfunction can also cause/exacerbate depression [28]. Prior research has revealed the high prevalence of depression among Chinese nurses [29], the present study further revealed the significant contribution of depression to SLS.

The Karasek-Demand-Control-Support (DCS) model is one of the most widely used models of job stress. The key idea behind this model is that control and social support buffer the negative effect of job demands on strain and therefore help maintain employees’ physical and mental health [30]. In keeping with this theory, we found high demands, low control and inadequate social support were significantly associated with low level of SLS of nurses. Importantly, because previous studies seldom focused on the relationship between job stress and the overall indicator of sex health, SLS, our findings extend the negative consequences of adverse workplace psychosocial factors from health outcomes to sex health. The association between high job stress and reduced SLS may be due to the suppression of the hypothalamic-pituitary- gonad axis, which is resulted from stress [12]. It is also possible that the declined SLS is a direct result of the job per se, for example, nurses have difficulties in maintaining a good partnership with sexual partners because of the busy job.

Finally, our analysis found that shift work and contract employment were significantly associated with low SLS of nurses, the former might be related to the abnormal sex hormone concentrations caused by biological rhythm disorders in shift workers [10, 11] and the latter might be due to the elevated level of depression of contract nurses [31, 32]. However, because depression and contract employment were simultaneously kept in the final model of multiple regression analysis, contract employment might exacerbate sexual dysfunction via mechanisms other than depressive emotion, which warrants further research.

Because sex live is the sexual contact between a man and a woman, sexual partner-related factors such as marital quality and relationship with sex partner also influence the SLS of nurses. However, data collection on sexual partner is not feasible in this survey, therefore we are not able to examine sexual partner-related factors associated with SLS of nurses. This is the major limitation of the current study. A second limitation is that some other risk factors of poor SLS such as life style, sleep quality, and anxiety were not assessed in this study so it is uncertain whether or not these factors would also be associated with decreased SLS of nurses. Third, the sample size of the survey was relatively small, thus limiting the generalizability of the findings. A final limitation is that this is a cross-sectional study so the factors we found associated with reduced SLS are not, strictly speaking, risk factors. Whether or not the identified factors cause reduction in SLS need to be determined by prospective longitudinal studies.

In summary, the present study demonstrated a relatively low level of SLS in Chinese nurses working in tertiary general hospitals and the reduced SLS is associated with certain demographic and adverse workplace psychosocial factors. Findings from the current study suggest that the SLS of nurses deserves special attention from nursing managers and hospital administrators. Reducing job stress, providing psycho-social support, and, when necessary, treating depression, may help improve SLS of Chinese nurses.

## MATERIALS AND METHODS

### Subjects

Between October 2016 and February 2017, this cross-sectional survey was conducted in four tertiary general hospitals of a metropolitan city in eastern China, Wenzhou. The four general hospitals were randomly selected from a total of ten city-owned tertiary general hospitals. By using the random number table, we further randomly invited 502 nurses (from a list of 1501 female nurses of the four hospitals) to participate in this study. Among these selected nurses, 473 agreed to join this study. The final sample for our analysis included 393 female nurses who self-reported having regular or irregular sexual partners and having had sex in the past month.

This study was approved by the Ethic Committee of Wenzhou Seventh People's Hospital. All participants provided written informed consent and completed the questionnaire anonymously.

### Measures

The self-administered questionnaire developed for this study consists of five major parts:

1. Socio-demographic questionnaire: age, education year, marital status, self-rated family income level (poor, moderate, and good), and having religious belief.

2. Occupational characteristics questionnaire: years of working, type of work (shift and regular day work), technical title (Nurse, Nurse Practitioner, Nurse-in-charge, Associate Professor of Nursing, and Professor of Nursing), and type of employment (contract and permanent staff).

3. The Chinese version of JCQ: The original JCQ is developed based on the DCS model [33], and has been one of the most widely used assessment of job stressors. The DCS model proposes that workers exposed to high levels of psychological demands, and low levels of job control and social support have high likelihood of suffering job stress. The Chinese JCQ has 22 items consisting of three subscales: psychological job demands (five items, workload and time pressure), job control (nine items, control over decisions and opportunity to use skills), and levels of work-related social support (eight items, support from coworkers and supervisors). Higher demands, control, and social support subscale scores indicate higher levels of psychological job demands, job control, and social support, respectively. Each item was recorded on a 4-point Likert scale, ranging from 1 (strongly disagree) to 4 (strongly agree). The Chinese JCQ is a reliable and valid instrument for measuring job stressors of Chinese working population [34]. In this study, Cronbach's α coefficients of the job demands, job control, and social support subscales of the Chinese JCQ were 0.75, 0.88, and 0.89, respectively.

4. The Chinese version of Zung's Self-rating Depression Scale (SDS): The SDS is a 20-item self-report questionnaire to assess depressive symptoms using a 4-point rating scale (1 = a little of the time to 4 = most of the time). Total score vary varies between 20 and 80, with higher scores demonstrating more symptoms of depression. A cut point of 40 or greater is defined as depression in Chinese population. The Chinese SDS has been proven to be reliable and valid for Chinese people [35]. The Cronbach's α coefficient of the Chinese SDS in our sample of nurses was 0.95.

5. SLS: SLS was assessed with a single question asking in the past month how satisfied the respondent is with her sex life with a 5-point Likert scale (1 = very dissatisfied; 2 = dissatisfied; 3 = fair; 4 = satisfied; 5 = very satisfied). This question is directly adapted from the World Health Organization Quality of Life Scale Brief Version (WHOQOL-BREF), which has been shown to be reliable and valid in Chinese population [36, 37]. This single-question measure has been widely employed in previous epidemiological studies of sex health [14, 15, 21, 24, 27].

### Statistical analysis

Socio-demographic and occupational characteristics and JCQ, SDS, and SLS scores were described, respectively. Although SLS scores in this study did not follow a normal distribution (skewness = 0.352, kurtosis = 0.655), we still used Two Independent Samples *t*-test to compare SLS scores between nurses of different characteristics. This is because the SLS scores are Likert data and a simulation study showed that, when using the two sample *t*-test and the Mann-Whitney test to analyze 5-point Likert items for two independent groups, both tests almost always provide the same protection against false negatives and always provide the same protection against false positives [38]. A second consideration for choosing *t*-test is that, because the mean (not median) of Likert scores can accurately represent the center of the distribution and the sample size of nurses is large enough (> 200), a parametric test should be more powerful [38]. Statistically significant factors in the *t*-tests were then entered together in multivariable ordinary logistic regression with a backward stepwise entry approach to identify factors of decreased SLS in nurses. ORs and 95% confidence intervals (CIs) were used to quantify the associations between factors and reduced SLS. The statistical significance level was set at *p* < 0.05 (two-sided). SPSS software version 16.0 package was used for analyses.
